# Lipidome-based rapid diagnosis with machine learning for detection of TGF-β signalling activated area in head and neck cancer

**DOI:** 10.1038/s41416-020-0732-y

**Published:** 2020-02-05

**Authors:** Hiroki Ishii, Masao Saitoh, Kaname Sakamoto, Kei Sakamoto, Daisuke Saigusa, Hirotake Kasai, Kei Ashizawa, Keiji Miyazawa, Sen Takeda, Keisuke Masuyama, Kentaro Yoshimura

**Affiliations:** 1 Department of Otolaryngology, Head and Neck Surgery, Chuo-city, Japan; 2 Center for Medical Education and Sciences, Chuo-city, Japan; 3 Section of Oral Pathology, Graduate School of Medical and Dental Sciences, Tokyo Medical and Dental University, Bunkyo City, Japan; 4 Department of Integrative Genomics, Tohoku University Tohoku Medical Megabank Organization, Sendai, Japan; 5 Department of Microbiology, Chuo-city, Japan; 6 Department of Biochemistry, Faculty of Medicine, University of Yamanashi, Chuo-city, Japan; 7 Department of Anatomy and Cell Biology, Faculty of Medicine, University of Yamanashi, Chuo-city, Japan

**Keywords:** Translational research, Surgical oncology, Oral cancer detection

## Abstract

**Background:**

Several pro-oncogenic signals, including transforming growth factor beta (TGF-β) signalling from tumour microenvironment, generate intratumoural phenotypic heterogeneity and result in tumour progression and treatment failure. However, the precise diagnosis for tumour areas containing subclones with cytokine-induced malignant properties remains clinically challenging.

**Methods:**

We established a rapid diagnostic system based on the combination of probe electrospray ionisation-mass spectrometry (PESI-MS) and machine learning without the aid of immunohistological and biochemical procedures to identify tumour areas with heterogeneous TGF-β signalling status in head and neck squamous cell carcinoma (HNSCC). A total of 240 and 90 mass spectra were obtained from TGF-β-unstimulated and -stimulated HNSCC cells, respectively, by PESI-MS and were used for the construction of a diagnostic system based on lipidome.

**Results:**

This discriminant algorithm achieved 98.79% accuracy in discrimination of TGF-β1-stimulated cells from untreated cells. In clinical human HNSCC tissues, this approach achieved determination of tumour areas with activated TGF-β signalling as efficiently as a conventional histopathological assessment using phosphorylated-SMAD2 staining. Furthermore, several altered peaks on mass spectra were identified as phosphatidylcholine species in TGF-β-stimulated HNSCC cells.

**Conclusions:**

This diagnostic system combined with PESI-MS and machine learning encourages us to clinically diagnose intratumoural phenotypic heterogeneity induced by TGF-β.

## Background

Tumour invasion and metastasis, and causes of treatment failure and death remain clinical issues.^1,2^ Advanced omics technologies targeting a single cell or a tumour bulk reveal the presence of tumour heterogeneity, characterised by phenotypic sub-clonality of tumour malignancy within a single tumour,^3–6^ which supports tumour invasion, metastasis, and treatment resistance in head and neck squamous cell carcinoma (HNSCC).^5,7^ Several extracellular cytokines are secreted locally from the tumour microenvironment to facilitate tumour invasion and metastasis through activating pro-oncogenic signalling pathways in tumour cells. Cytokine-mediated extracellular signals are both spatial and temporal within a single tumour, resulting in different subclones of tumour cells showing different responses to these signals.^8,9^ Therefore, intratumoural phenotypic heterogeneity affects the clinical determination of tumour malignancy and potentially exerts a harmful influence on treatment decisions.

Transforming growth factor beta (TGF-β), a critical mediator in the induction of the epithelial–mesenchymal transition (EMT) and in the development of the tumour microenvironment, renders tumour cells more invasive and metastatic.^10^ Different concentrations of TGF-β1 ligand generate intratumoural heterogeneity of TGF-β1 signalling at the tumour–stroma interface, and increase the pool of TGF-β1-responding progenitor cells responsible for drug resistance and tumour recurrence in SCC.^11,12^ Recent studies have suggested that tumour cells with a partially activated EMT program localize to the leading edge of primary tumours, and a partial EMT results in phenotypic diversity within a single tumour.^5,13^ Therefore, precise diagnosis of intratumoural heterogeneity of tumour cells with a different TGF-β signalling status needs to be achieved for better clinical decisions regarding appropriate treatments for tumour invasion and metastasis. However, accumulating evidences have had little impact on the establishment of diagnoses targeting intratumoural phenotypic heterogeneity at the bedside.

Ambient ionisation methods combined with mass spectrometry (MS), such as desorption electrospray ionisation-MS,^14^ rapid evaporative ionisation-MS,^15^ and probe electrospray ionisation (PESI)-MS, are powerful tools for the rapid analysis of biological molecules. Especially PESI-MS enables acquisition of mass spectra showing profiles of lipid metabolites from a small amount of biological sample (10 mg) with minimal invasiveness and pre-treatment.^16,17^ The lipidome is informative in the acquisition of malignant phenotypes of tumour cells.^18^ Importantly, we explored the intraoperative diagnosis system using PESI-MS combined with machine learning to discriminate cancerous regions from non-cancerous regions in HNSCC.^19^ This approach not only precisely determines a tumour border between non-cancerous and cancerous regions, as assessed by pathologists, but also reveals lipid metabolic heterogeneity in HNSCC tissues. Previous reports have shown that activation of malignant signalling directly influences lipid metabolism in tumour cells, but it remains unclear as to whether PESI-MS could characterise alterations in lipid metabolites in tumour cells stimulated with TGF-β1, and whether it is applicable in determining regions containing TGF-β1-stimulated tumour cells.

Here we established a rapid diagnostic system for determining heterogenous TGF-β signalling status within a single HNSCC tumour, based on PESI-MS and machine learning. To construct a diagnostic algorithm for tumour areas stimulated with TGF-β1 in HNSCC, we acquired a total of 240 and 90 mass spectra from human recombinant TGF-β1-stimulated or -unstimulated human HNSCC cells, respectively, using both the positive- and negative-ion modes, and then logistic regression (LR) was used for discrimination analysis as a diagnostic algorithm. To confirm the usability of our diagnostic system in clinical settings, we sought to discriminate tumour areas with activated TGF-β signalling within a HNSCC tissue. Moreover, lipid characterisation of HNSCC cells stimulated with TGF-β1 revealed an increase in phosphatidylcholine (PC) species with an alteration in lysophosphatidylcholine acyltransferase 2 (*LPCAT2*) expression. Taken together, our data suggest that this system using PESI-MS and machine learning allows us to rapidly diagnose tumour areas with heterogenous TGF-β signalling status within a HNSCC tissue. This achievement could contribute to better treatment decisions for HNSCC patients.

## Materials and methods

### Cell lines and cell viability assay

Seven human HNSCC cell lines (SAS, HSC2, HSC3, HSC4, Ca9-22, KUMA1, and Gun-1) were cultured as described previously.^20^ All human HNSCC cell lines were authenticated by short tandem repeat (STR) profiling. Cells were stimulated in vitro for 24–48 h with 2 ng/ml recombinant TGF-β1 and dimethyl sulfoxide as a control. For viability assays, the number of TGF-β1-stimulated HNSCC cells was evaluated 48 h after treatment with 10 µM docetaxel (DTX) (Sigma-Aldrich) or 15 µM cisplatin (CDDP) (Sigma-Aldrich) by cell counter and their viability was determined by scoring of Trypan blue uptake.

### Quantitative RT-PCR

Total RNA was extracted from cells using the RNeasy Mini Kit (Qiagen) and then reverse transcribed into complementary DNA (cDNA) using the SuperScript VILO cDNA synthesis kit (Invitrogen). Quantitative RT-PCR (RT-qPCR) was performed using SYBR Green Master and the ABI7300 real-time PCR system (Applied Biosystems). Each mRNA was normalised to *GAPDH* mRNA levels. The relative expression levels of targeted genes were calculated by the 2^−ΔΔCt^ method.

### Immunoblotting analysis

Protein extracts from HNSCC cells were analysed by immunoblotting as previously described.^20^ The following primary antibodies were used: human phospho-SMAD2 (1:1000, AB3849; Millipore Sigma), SMAD (1:1000, 138D4; Cell signalling Technology), LPCAT2 (1:1000, NBP1-88921; Novus Biologicals), and α-tubulin (1:2000, DM1A; Sigma-Aldrich). Anti-mouse/rabbit/goat secondary antibodies were purchased from Jackson ImmunoResearch Laboratories (1:2000). Blots of each protein were visualised using Amersham Bioscience ECL Western blotting detection reagent (GE-Healthcare). All images were acquired with a LAS-4000 mini imager (Fujifilm) and protein bands were also quantified by Image Reader LAS-4000 software (Fujifilm).

### Wound healing assay

Cells were inoculated in 6-well plates. After cells reached confluency, they were scratched with a 10-µl disposable pipette tip. Migration of wound edges was measured at five random points on images acquired using an ECLIPSE TS100 microscope (Nikon), and the cell migration distance after 10 h was compared with the distance at 0 h. Data analysis was performed according to the previous protocol.^20^

### Study approval and immunohistochemistry

Oral tissues were fixed by neutral-buffered formalin and embedded in paraffin to assess SMAD2 phosphorylation. After preparation of thin sections, slides were deparaffinised with xylene, followed by rehydration through graded alcohol to water. Antigen retrieval was performed in target retrieval solution (pH 9.0) at 120 °C for 15–20 min (S2367;  Dako). Then, endogenous peroxidase was quenched with 3% (v/v) H_2_O_2_ for 5 min. After blocking with 1% bovine serum albumin (BSA) at room temperature for 2 h, the sections were incubated with a primary antibody diluted with 1% BSA in phosphate-buffered saline (PBS). Slides were incubated overnight at 4 °C with primary monoclonal antibodies. Primary antibody was as follows: anti-phospho-SMAD2 (1:100, 3108; Cell signalling Technology). Slides were then incubated with horse radish peroxidase using a ChemMate EnVision kit (Dako) for 2 h and washed twice with PBS. Immunoreactivity was visualised with 0.6 nm 3,3′-diaminobenzidine tetrahydrochloride (Dojindo) and counterstained with haematoxylin. Images were acquired with a BX53 microscope and DP72 microscope digital camera (Olympus) and analysed using Olympus cellSens software. ImageJ software was used to quantify phosphorylated-SMAD2 (p-SMAD2) protein levels.

### Probe electrospray ionisation-mass spectrometry

Cells were scraped with a Cell Scraper 16 cm 2-Pos.-Blade (SARSTEDT AG & Co.) and centrifuged at 800 × *g* for 5 min. Ten microliters of cell pellets were suspended with 200 µl of 50% ethanol to extract cellular molecules and denature the proteins. After centrifugation at 15,000 × *g* for 5 min, 85 µl of supernatants were analysed by PESI-MS (DPiMS-2020, Shimadzu). The procedure for preparation of human tissues and analysis by PESI-MS was described in our previous report.^19^ Analyses were performed in positive- and negative-ion modes for each sample. Five representative mass spectra from each analysis were generated using the LabSolutions software (ver. 5.82 SP1; Shimadzu). The procedures for data processing, analysis, and discrimination by partial least-squares-LR (PLS-LR) were described in our previous study.^19^

### Liquid chromatography-Fourier transform MS

Total lipid from 5 µl of SAS cell pellet was extracted according to the method of Bligh and Dyer. Dried total lipid was dissolved in 200 µl of 0.1% formic acid in methanol. After dissolving, this solution was further homogenised by ultrasonic bath for 5 min. Sixty microliters of the resultant solution was transferred to the vial, and 4 µl of solution was injected for each analysis.

Liquid chromatography-Fourier transform MS (LC-FTMS) analysis was performed on a NANOSPACE SI-II HPLC (Osaka Soda) system coupled with a Q Exactive Orbitrap MS (Thermo Fisher Scientific) equipped with heated electrospray ionisation-II probe operated in positive-ion mode. The mass resolution was 70,000 and set to acquire data over an *m/z* range from 280 to 1000. LC separation was performed using a reverse phase column (L-column 2 C8, 50 mm × 2.1 mm i.d., 3.0 µm particle size; CERI) with a gradient solution of solvent A (water containing 5 mM ammonium formate, pH 4.0) and B (95% acetonitrile in water containing 5 mM ammonium formate, pH 4.0) at 0.2 mL/min. Solvent A was prepared by mixing 99.5 mL of Milli-Q (Millipore) water and 0.5 mL of 1 M ammonium formate (Fujifilm), and adjusting to pH 4.0 with formic acid (9.6 µL, Fujifilm). Solvent B was prepared by mixing 95 mL of acetonitrile (Kanto Kagaku), 4.5 mL of Milli-Q water, and 0.5 mL of 1 M ammonium formate, and adjusting to pH 4.0 with formic acid (~1,160 µL). The initial condition was set at 30% B. The following solvent gradient was applied: 30% B for 2 min, a linear gradient to 100% B from 2 to 20 min, and then 100% B for 10 min. Subsequently, the mobile phase was immediately returned to the initial conditions and maintained for 5 min until the end of the run. The oven temperature was 40 °C. Data processing and molecular identification were performed according to a previous report.^21^

### Correlative studies with public datasets

Publicly available datasets of human squamous cell carcinoma cell lines stimulated with or without TGFβ1 (GSE57441) were used to identify differentially expressed genes that were induced by TGFβ1 stimulation. For Kaplan–Meier survival curve analysis in HNSCC patients, TCGA dataset was used. The correlations of *LPCAT2* gene expression levels with tumour progression and distant metastasis-free survival were analysed based on publicly available datasets of human oral cancer tissues (GSE9844).

### Statistical analysis

GraphPad Prism (8.2.1) was used for graphs and for statistics. Unless otherwise indicated, all data were analysed using the Student’s *t* test and are expressed as mean ± SD. Kaplan–Meier analysis and log-rank tests were applied for survival analysis. Differences were considered statistically significant when *p* < 0.05.

## Results

### Activated TGF-β signalling enhances cell motility and increases resistance to chemotherapeutic agents in HNSCC cells

**Fig. 1:**
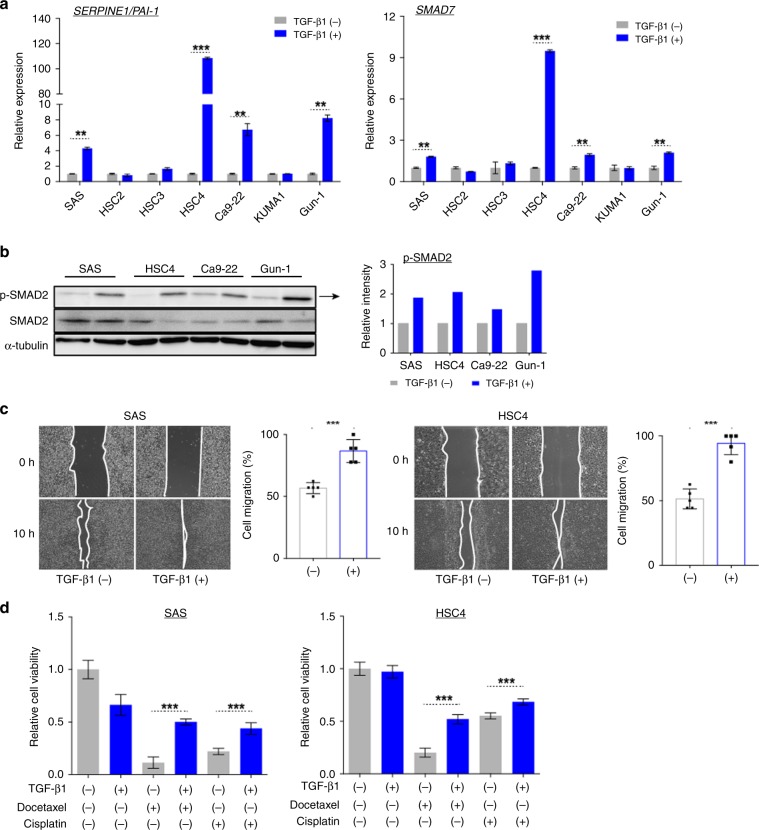
Activated TGF-β signalling enhances cell motility and increases chemoresistance in HNSCC cells. **a** RT-qPCR for *PAI-1* (left) and *SMAD7* (right) mRNA expression in seven different HNSCC cell lines that were unstimulated or stimulated with 2 ng/ml human recombinant TGF-β1 for 48 h. Each value was normalised to *GAPDH* mRNA expression. **b** Phospho-SMAD2 (p-SMAD2) and SMAD2 protein levels in the indicated HNSCC cells stimulated with or without 2 ng/ml TGFβ1 for 48 h. α-tubulin was used as a loading control for immunoblotting. Right panel shows quantification of p-SMAD2 normalised against α-tubulin expression. **c** Wound healing assay of SAS and HSC4 cells cultured with or without 2 ng/ml TGF-β1 for 48 h. Cell migration distances after 10 h at five randomly chosen points were measured and compared with the distances at 0 h. **d** Cell viability assays in TGF-β1-unstimulated or -stimulated SAS (left) and HSC4 (right) cells that were exposed to dimethyl sulfoxide (DMSO), docetaxel, or cisplatin. The data are represented as mean ± SEM. All *p* values were determined by Student’s *t* test. **p < 0.01 and ***p < 0.001.

TGF-β signalling increases the expression of genes such as *SERPINE1/PAI-1*, *SMAD7*, and *VIM* by activating the canonical SMAD pathway. We first confirmed that recombinant human TGF-β treatment induces activation of the canonical TGF-β/SMAD pathway in seven HNSCC cell lines, that is, SAS, HSC2, HSC3, HSC4, Ca9-22, KUMA1, and Gun-1 cells. In SAS, HSC4, Ca9-22, and Gun-1 cells, *SERPINE1/PAI-1*, *SMAD7*, and *VIM* expression was significantly upregulated when stimulated with recombinant human TGF-β1 (Fig. 1a and Supplementary Fig. 1a). SMAD2 was also adequately phosphorylated in these cells (Fig. 1b). We further evaluated whether HNSCC cells with activated TGF-β signalling acquired malignant properties. Wound closure was faster in HNSCC cells stimulated with TGF-β1 than naive control cells (Fig. 1c and Supplementary Fig. 1b), indicating that activation of the TGF-β signalling pathway facilitates high cell motility of HNSCC cells. To examine whether TGF-β1 stimulation facilitates acquisition of chemoresistance in HNSCC cells, cell viability was evaluated by DTX or CDDP treatment in SAS, HSC4, Ca9-22, and Gun-1 cells stimulated with TGF-β1 (Fig. 1d). This treatment alone slightly inhibited cell proliferation in only SAS cells, but not in HSC4, Ca9-22, and Gun-1 cells without DTX or CDDP treatment (Fig. 1d and Supplementary Fig. 1c). Compared with unstimulated SAS, HSC4, Ca9-22, and Gun-1 cells, TGF-β1-stimulated cells showed more resistance against DTX (10 µM) and CDDP (15 µM) (Fig. 1d and Supplementary Fig. 1c). Taken together, these findings indicate that activation of the TGF-β signalling pathway contributes to acquisition of malignant properties in HNSCC cells.

### Construction of a discriminant algorithm for determining activation of TGF-β signalling in HNSCC cells using ambient MS and machine learning

Tumor cells show a distinctive metabolic profile from healthy epithelial cells, and especially, alterations in lipid metabolism are observed in tumour cells that have acquired malignant phenotypes.^18,22,23^ Our previous study reported that, based on unique metabolome profiles in cancer tissues, PESI-MS allows us to discriminate cancerous regions from non-cancerous regions.^19,24,25^ Moreover, TGF-β/SMAD signalling is associated with phospholipid metabolism in liver injury;^26^ however, the effects of TGF-β on lipid metabolism remain unclear in tumour tissues.

**Fig. 2:**
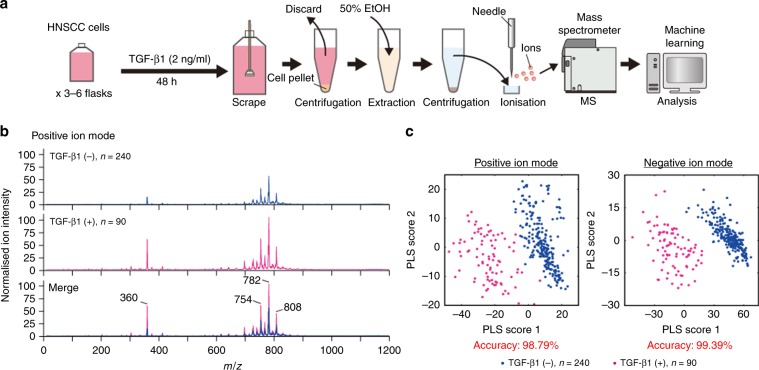
Construction of a discriminant algorithm for determining activation of TGF-β signalling in HNSCC cells. **a** The procedures of sample preparation for probe electrospray ionisation-mass spectrometry (PESI-MS) and the predictive system for activated TGF-β signalling in HNSCC cells by machine learning. **b** Mean normalised mass spectra (*m/z* 10–1200) of HNSCC cells that were unstimulated (upper: *n* = 240) or stimulated with 2 ng/ml TGF-β1 for 48 h (middle: *n* = 90) in positive-ion mode. Merged mass spectra are shown in the bottom panel for comparisons between TGF-β1-unstimulated and TGF-β1-stimulated HNSCC cells. **c** Predictive accuracy of the diagnostic system based on PESI-MS and partial least-squares (PLS)-logistic regression algorithm for activation of TGF-β signalling in HNSCC. Plots of the first two PLS scores are shown for the mass spectra from positive- (left) or negative- (right) ion mode. The accuracy is the percentage of correctly classified mass spectra.

To explore whether activation of TGF-β signalling affects lipidome alteration in HNSCC cells, we first analysed lipidome in recombinant human TGF-β1-unstimulated or -stimulated HNSCC cells. As shown in Fig. 2a, biological extracts were prepared from TGF-β1-unstimulated or -stimulated HNSCC cells and analysed by PESI-MS. A total of 240 and 90 mass spectra were acquired from TGF-β1-unstimulated and -stimulated HNSCC cells, respectively. The analyses were performed in both positive- and negative-ion modes to obtain the mass spectra (Fig. 2b, Supplementary Fig. 2a). Ion peaks were mainly detected in two distinct regions, within a range of *m/z* 300–450 and 700–850, on mass spectra from both the positive- and negative-ion modes. Based on statistical analysis by Student’s independent two-sample *t* test of each peak, we identified TGF-β-activity-related peaks in positive-ion mode (Supplementary Fig. 2b), indicating that PESI-MS showed significant differences in lipidome between TGF-β1-unstimulated and -stimulated HNSCC cells.

We next sought to develop a new diagnostic system based on lipidome in HNSCC cells to identify tumour areas containing HNSCC cells stimulated with TGF-β1 within a single tumour. First, a diagnostic system using PESI-MS and a machine learning-based discriminant algorithm was constructed. Mass spectra from human recombinant TGF-β1-unstimulated and -stimulated HNSCC cells were visually distinct on PLS score plots of positive- and negative-ion modes (Fig. 2c). All mass spectra were used for learning of PLS-LR, and we validated the accuracy of the discriminant algorithms by performing leave-one-out cross-validation. The accuracy rates were 98.79% and 99.39% in the positive- and negative-ion modes, respectively (Fig. 2c). Of note, the process from sample preparation to obtaining a diagnosis for activated TGF-β signalling required 5 min. Taken together, our algorithm precisely discriminated human HNSCC cells stimulated with TGF-β1 from untreated control cells in vitro.

### In vivo rapid diagnosis of tumour areas containing tumour cells with high p-SMAD2 levels within clinical HNSCC tissues

**Fig. 3:**
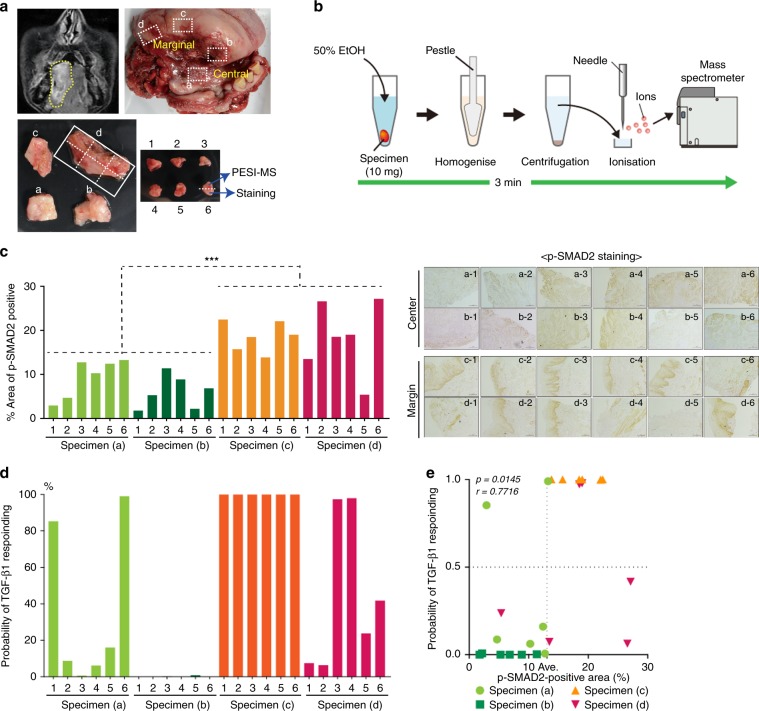
In vivo lipidome-based rapid diagnosis of tumour areas stimulated with TGF-β1 in human HNSCC tissues. **a** Magnetic resonance image of advanced oral SCC tissues (upper-left). The area surrounded by a yellow dash line shows a tumour lesion. The sample preparation for a test of the diagnostic system for activation of TGF-β signalling in HNSCC (upper-right, lower-left, and right). Major specimens (**a**, **b**) or specimens (**c**, **d**) were excised from a central or marginal area of human HNSCC tissue, respectively. **b** The procedures of sample preparation for PESI-MS. **c** Left panels: quantification of p-SMAD2-positive areas in specimens from a central or marginal area of HNSCC tissues. *X*-axis represents the percentage of p-SMAD2-positive areas on the images from specimens. *P* values were determined by Student’s *t* test. ***p < 0.001. Right panels: All representative images of immunohistochemistry for p-SMAD2 (brown) in specimens from a central and marginal area of HNSCC. Scale bar: 100 µm. **d** The probability of activated TGF-β signalling in each specimen from a central or marginal area of HNSCC tissue. A test spectrum was assigned to the class with the higher posterior probability. **e** Spearman’s analysis of the correlation between the probability of activated TGF-β signalling and p-SMAD2-positive areas in specimens from a central or marginal area of HNSCC tissue. *r*, Pearson’s correlation coefficient.

To confirm the clinical utility of our rapid diagnostic system at the bedside, this system was further tested on surgically resected tumour tissues from a patient with advanced HNSCC. As shown in Fig. 3a, ~5 mm^3^ of four representative tissues (a–d) were collected from central and marginal areas of oral SCC tissue. Each tissue was further separated into six specimens and analysed by both PESI-MS and immunohistochemistry (IHC) for p-SMAD2, a conventional examination to evaluate activation of TGF-β signalling and identify HNSCC cells stimulated with TGF-β within a tumour tissue (Fig. 3b and Supplementary Fig. 3). As reported in a previous study evaluating TGF-β signalling between marginal and central areas within tumour tissue,^27^ the expression levels of p-SMAD2 were significantly higher in tumour cells located at marginal tumour areas than those at central tumour areas (Fig. 3c), indicating that HNSCC cells located at marginal tumour areas showed more response to TGF-β1 stimulation than central areas. Importantly, there was a distribution of tumour cells stimulated with TGF-β1 within a HNSCC tissue.

Next, mass spectra were obtained in positive-ion mode from the same tumour specimens in which p-SMAD2 expression was evaluated by IHC. Based on the mass spectra from tumour specimens, our discriminant algorithm calculated the results in each dissected tumour area and predicted that the marginal tumour areas contained more HNSCC cells with high p-SMAD2 levels than the central tumour areas of HNSCC tissue (Fig. 3d and Table 1). We also examined the corresponding data between the predicted result obtained by machine learning and p-SMAD2 expression levels in each dissected tumour area. Predicted results from our discriminant algorithm corresponded with pathological assessment of high p-SMAD2 levels in HNSCC tissues (Fig. 3e). It strongly suggests that our system is applicable to in vivo detection of tumour areas in which HNSCC cells show activation of TGF-β signalling.

**Table 1:** Probabilities in central-area and marginal-area of HNSCC tissue.

Central area			Marginal area		
Specimen	Inactivated	Activated	Specimen	Inactivated	Activated
(a)-1	0.1471	**0.8529**	(c)-1	0.0000	**1.0000**
(a)-2	**0.9133**	0.0867	(c)-2	0.0000	**1.0000**
(a)-3	**0.9938**	0.0062	(c)-3	0.0001	**0.9999**
(a)-4	**0.9382**	0.0618	(c)-4	0.0000	**1.0000**
(a)-5	**0.8398**	0.1602	(c)-5	0.0011	**0.9989**
(a)-6	0.0098	**0.9902**	(c)-6	0.0000	**1.0000**
(b)-1	**0.9998**	0.0002	(d)-1	**0.9264**	0.0736
(b)-2	**1.0000**	0.0000	(d)-2	**0.9372**	0.0628
(b)-3	**0.9983**	0.0017	(d)-3	0.0271	**0.9729**
(b)-4	**0.9997**	0.0003	(d)-4	0.0211	**0.9789**
(b)-5	**0.9921**	0.0079	(d)-5	**0.7628**	0.2372
(b)-6	**1.0000**	0.0000	(d)-6	**0.5829**	0.4171

Bold face values show high number of probability in each specimen.

### TGF-β signal activation induces reprogramming of lipidome in cultured HNSCC cells

**Fig. 4:**
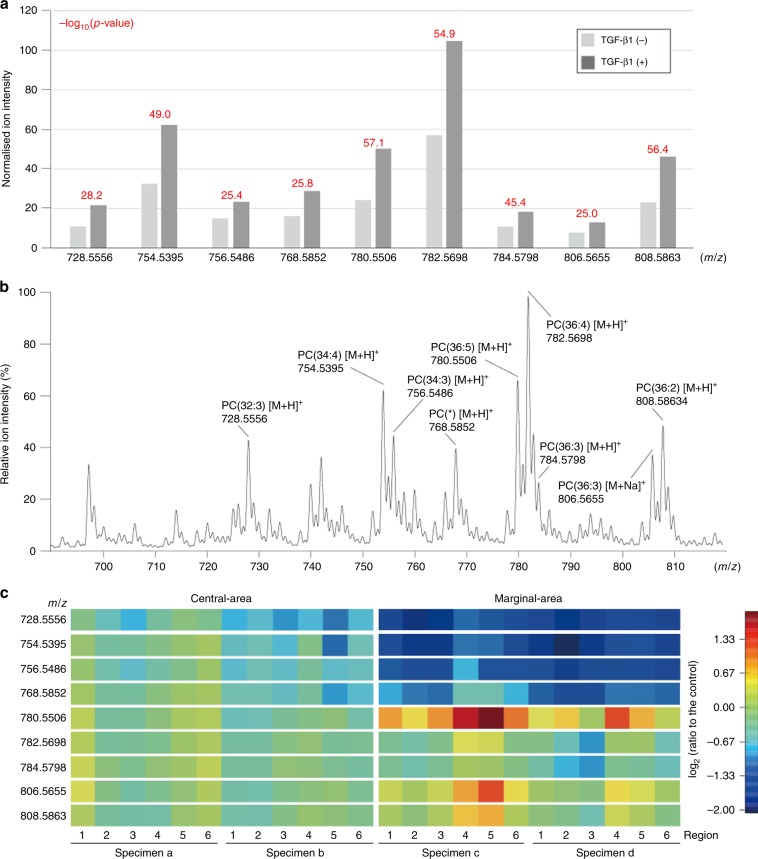
TGF-β stimulation enriches phosphatidylcholine contents in HNSCC cells. **a** Normalised ion intensity of each phosphatidylcholine (PC) species determined by LC-MS/MS methods in PESI-MS mass spectra from TGF-β1-unstimulated or -stimulated SAS cells. Each number above the graphs indicates −log_10_(*p*-value) of statistical comparisons between TGF-β1-unstimulated or -stimulated SAS cells. **b** Identification of PC species by liquid chromatography with tandem MS (LC-MS/MS) method in SAS cells that were stimulated with 2 ng/ml TGF-β1 for 48 h. The molecular species are annotated on a mass spectrum. (*): PC with alkylether (36:4) or plasmalogen (36:3) at sn-1 position. **c** Relative ion intensities of specific peaks representing PC species, which were determined in **a**, in specimens from a central or marginal area of HNSCC tissue are here presented as heatmap images. Y-axis indicates the *m/z* of each PC peak and X-axis indicates the number of each specimen as shown in Fig. 3a.

Following our initial analysis of significantly altered metabolites in TGF-β1-stimulated versus -unstimulated HNSCC cells, TGF-β-induced lipidome alteration was next examined in HNSCC cells. We then ranked metabolites in increasing order of *p* value, which were calculated using Student’s *t* test, to identify the most significantly altered metabolites between TGF-β1-stimulated and -unstimulated SAS cells. As shown in Fig. 4a, there were nine representative increased peaks with high *p* values [–log_10_(*p*-value) >25.0] on mass spectra from positive-ion mode in TGF-β1-stimulated SAS cells. Additionally, using LC-FTMS, we identified PC species PC(32:3), (34:4), (34:3), (36:5), (36:4), (36:3), (36:3), (36:2), and PC with alkylether (36:4) or plasmalogen (36:3) at sn-1 position (Fig. 4b). Taken together, these increased PC species have the potential to be biomarkers to clarify HNSCC cells stimulated with TGF-β1.

To further investigate whether these top-ranked metabolites were consistently increased in clinical tumour areas where TGF-β signalling was activated, we analysed the ion intensities of nine PC species in clinical HNSCC tissues. PC(36:5), (36:3), and (36:2) were increased in marginal tumour areas containing HNSCC cells with high p-SMAD2 levels. Conversely, PC(32:3), (34:4), (34:3), and PC with alkylether (36:4) or plasmalogen (36:3) at sn-1 position were decreased in marginal areas (Fig. 4c), indicating that some of the altered PC species represent potentially important metabolic features in HNSCC cells with activated TGF-β signalling and are surrogate lipid markers for activation of TGF-β signalling in HNSCC.

### Increased LPCAT2 is associated with metastasis in SCC patients

**Fig. 5:**
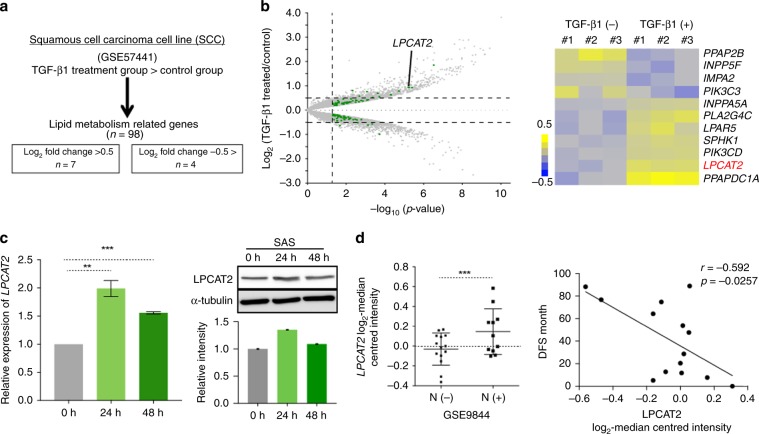
*LPCAT2* correlates with lymph node metastases and decreased survival in SCC patients. **a** Strategies to identify differentially expressed genes related to lipid metabolism in SCC cells that were stimulated with 2 ng/ml human recombinant TGF-β1 for 48 h. A publicly available dataset (GSE57441) was used for this analysis. **b** Left: identification of significantly increased (log_2_ FC > 0.5) or decreased (log_2_ FC < 0.5) genes related to lipid metabolism in TGF-β1-stimulated SCC cells. Right: summarised heatmap for expression of each gene related to lipid metabolism between TGF-β1-unstimulated (*n* = 3) or -stimulated (*n* = 3) SCC cells. **c** RT-qPCR (left) and immunoblotting (right) for expression of *LPCAT2* mRNA and protein in SAS cells that were unstimulated or stimulated with 2 ng/ml TGF-β1 for 24 and 48 h. Each value was normalised to *GAPDH* mRNA expression, and α-tubulin was used as a loading control for immunoblotting. Right lower panel shows quantification of LPCAT2 normalised against α-tubulin expression. **d** *LPCAT2* correlates with lymph node metastatic status (left) and distant metastasis-free survival of HNSCC patients (right). Publicly available datasets (GSE9844 and GSE75538) were used for the analysis. The log_2_ median-centred intensity of *LPCAT2* expression was compared between HNSCC patients with (*n* = 14) or without (*n* = 11) lymph node metastasis (left). Spearman’s analysis of the correlation between *LPCAT2* expression and distant metastasis-free survival in HNSCC patients (*n* = 14) is shown in the right panel. *r*, Pearson’s correlation coefficient.

To elucidate the mechanisms responsible for lipidome including increased PC species in TGF-β1-stimulated HNSCC cells, we next attempted to identify differentially expressed genes associated with lipidome alteration in TGF-β1-stimulated SAS cells compared with unstimulated SAS cells. A publicly available database of whole transcriptome data (GSE57441) where SCC cells established from uterine cervix were stimulated with 2 ng/ml human recombinant TGF-β1 for 48 h was used for identification of metabolic regulators. A total of 98 lipid metabolism-related genes were identified on this dataset (Fig. 5a). Among these, there were seven upregulated genes and four downregulated genes in TGF-β1-stimulated SCC cells (Fig. 5b). Interestingly, *LPCAT2*, the product of which catalyses the conversion of lysophosphatidylcholine to PC,^28^ was significantly increased in TGF-β1-stimulated SCC cells (Fig. 5b). Indeed, we verified that the mRNA and protein expression levels of *LPCAT2* were higher in TGF-β1-stimulated SAS cells than those in TGF-β1-unstimulated SAS cells (Fig. 5c).

Previous studies of LPCAT enzymes in cancer have shown that they are independent predictors of early tumour recurrence and prognostic markers for several cancers.^29–31^ Particularly, the expression level of LPCAT2 was positively correlated with aggressive prostate cancer;^32^ however, the impact of increased *LPCAT2* expression on clinical progression remains unknown in HNSCC patients. We used publicly available data from patients with HNSCC (GSE9844 and GSE75538) to investigate the clinical relevance of *LPCAT2* expression in metastasis and survival of HNSCC patients. Although *LPCAT2* expression in HNSCC tissues did not significantly affect overall survival (Supplementary Fig. 4), increased *LPCAT2* levels were found in HNSCC patients with lymph node metastases compared with HNSCC patients without lymph node metastases (Fig. 5d, left panel). In addition, *LPCAT2* expression was negatively correlated with distant metastasis-free survival (Fig. 5d, right panel). Therefore, increased *LPCAT2* is potentially relevant to metastasis of HNSCC.

## Discussion

Accumulating evidences in the field of intratumoural heterogeneity have created a new trend in clinical cancer diagnosis and therapeutic strategies. To accomplish more personalised and better cancer treatment decisions at the bedside, it is important to diagnose intratumoural phenotypic heterogeneity where protumoral signalling is temporally and spatially activated within a single tumour. In recent years, diagnostic pathology for protumoral signalling was exclusively realised using only a few predictive markers such as epidermal growth factor receptor and human epidermal growth factor receptor 2) in the clinic. However, these assessments are still retrospective and limited because tumour samples need to be formalin fixed and paraffin embedded to enable staining of the representative downstream molecules by specific antibodies. Moreover, only a few markers can precisely evaluate activation of complicated downstream signalling cascades in a single tumour. Therefore, precise detection and diagnosis for intratumoural heterogeneity of activated protumoral signalling pathways in cancer tissues remain challenging. Especially, few downstream markers are used for evaluation of activated TGF-β signalling in tumour tissues instead of p-SMAD2 IHC.

Here, we described a rapid diagnostic system based on combination of PESI-MS with machine learning to detect tumour areas stimulated with TGF-β1 in clinical HNSCC tissue. As shown in Fig. 2b, PESI-MS rapidly captured distinct metabolic profiles in human recombinant TGF-β-stimulated HNSCC cells and provided discriminative spectral patterns of TGF-β-stimulated HNSCC cells on mass spectra. Additionally, it enabled the acquisition of mass spectra data from a small number of biological components using a very fine needle with minimum invasiveness in a clinical situation where it might be difficult to undertake diagnosis by IHC. It takes ~5 min to obtain a mass spectrum from a sample. The properties of PESI-MS are also of advantage to reveal spatially and temporally phenotypic heterogeneity within a single tumour compared with conventional pathological diagnosis using IHC.

To further determine whether this system could be applied to clinical and pathological diagnosis of tumour areas containing HNSCC cells stimulated with TGF-β1 at the bedside, we used machine learning-based discriminant algorithm established by using a large number of mass spectra from TGF-β1-stimulated and -unstimulated HNSCC cells. Machine learning is authorised as a powerful tool of algorithms to facilitate pattern recognition, classification, and prediction based on statistical models using existing data.^33^ Recently, clinical diagnosis combined with machine learning has been broadly used and can be applied to the identification of biomarkers in breast cancer.^34^ We recently demonstrated that a diagnostic system based on PESI-MS and a machine-learning algorithm can discriminate cancerous regions from non-cancerous regions and can achieve a rapid intraoperative assessment of tumour margins in head and neck cancer.^19^ Current discriminant algorithms learning mass spectra in this study can enable biological determination in cultured cells activated by TGF-β signalling pathway without conventional analyses of downstream molecules with over 98% accuracy. In in vivo assessments using human HNSCC tissues, predictive results associated with activation of TGF-β signalling in HNSCC tissue had a positive correlation with p-SMAD2 staining intensity within HNSCC tissues. Of note, this system determined the areas containing only stromal components such as muscles, fat, and salivary glands (d-1 and d-5 in Fig. 3c) in which p-SMAD2 staining was positive as areas without activation of TGF-β signalling. In contrast, the areas in which only a little epithelial component with p-SMAD2 positive staining was contaminated (d-4 in Fig. 3c) was diagnosed as areas with activation of TGF-β signalling. Our diagnostic system would reduce false positives and accurately provide more specific diagnoses of tumour areas containing tumour cells with activated TGF-β signalling. Especially given that the system provided high probabilities of activated TGF-β signalling in some areas containing tumour cells with less p-SMAD2 (a-1 and a-6 in Fig. 3c), our diagnostic system could identify the possibility that tumour cells in those areas have acquired malignant phenotypes induced by TGF-β. If tumour areas containing TGF-β1-stimulated tumour cells are determined during surgery, surgeons could design a more personalised tumour margin dissection for each patient. Moreover, in order to make better clinical decisions regarding appropriate treatments for HNSCC, identification of tumour areas with activated TGF-β signalling pathway would help pathologist to assess clinical risks for metastasis, recurrence, and chemoresistance in HNSCC. However, we further need to seek out the clinical meaning of positive diagnoses for determining HNSCC areas containing tumour cells with activated TGF-β signalling.

In our current study, PESI-MS and liquid chromatography-combined tandem MS revealed increased PC species that are relevant for the activation of TGF-β signalling. Correspondingly, *LPCAT2* was also increased in these cells and was recognised as a biomarker of tumour metastasis in HNSCC. Other recent studies have shown that tumour cells have aberrant *de novo* lipogenesis to acquire malignant phenotypes such as proliferation, high motility, or chemoresistance, and lipidome alteration is recognised as a risk of cancer progression and metastasis.^18,35^ LPCAT2 is also responsible for lipid droplet accumulation in colorectal cancer cells and supports chemoresistance.^36^ Considering these evidences supporting our data, TGF-β1 can cause lipid metabolic reprogramming in HNSCC cells, and TGF-β1-induced *LPCAT2* might be responsible for chemoresistance and high-motility in HNSCC cells through lipid metabolic reprogramming including PC accumulation. We also focused on top-ranked metabolic features, which might have implications for activation of TGF-β signalling, leading to acquisition of malignant phenotypes in HNSCC. However, it is unclear whether these top-ranked PC species predominantly drive the phenotypic differences in HNSCC cells with or without activated TGF-β signalling. Of note, our study also clarified “decreased PC species” in cultured cells or tumour areas with activated TGF-β signalling, and revealed that phospholipase A2, an enzyme that decreases the abundance of PC species, was also increased in SCC cells stimulated with TGF-β1 (Fig. 5b). These observations might indicate complicated phospholipid alterations in HNSCC. The exact mechanisms responsible for lipid metabolic reprogramming induced by TGF-β1 stimulation in HNSCC cells will be the focus of a subsequent study.

In conclusion, our study strongly indicates that a diagnostic system based on combination of PESI-MS and a machine learning-based algorithm can be used to assist the rapid detection of tumour areas with activated TGF-β signalling in HNSCC tumour tissue without conventional immunohistological examination, and has the potential to be a feasible method to diagnose intratumoural phenotypic heterogeneity of TGF-β signalling at the bedside.

## Supplementary information

Supplemental information

## Data Availability

The data supporting the findings of this study are available within the article and its Supplementary information file or are available from the corresponding author upon request.
