# Supplementary material for: Lipidome-based rapid diagnosis with machine learning for detection of TGF-β signalling activated area in head and neck cancer
Source: Br J Cancer. 2020 Feb 5;122(7):995–1004. doi: 10.1038/s41416-020-0732-y (PMC7109155; doi:10.1038/s41416-020-0732-y)
Supplement: Supplementary file 1 — Supplemental information [file 41416_2020_732_MOESM1_ESM.pdf]

Supplementary figure 1

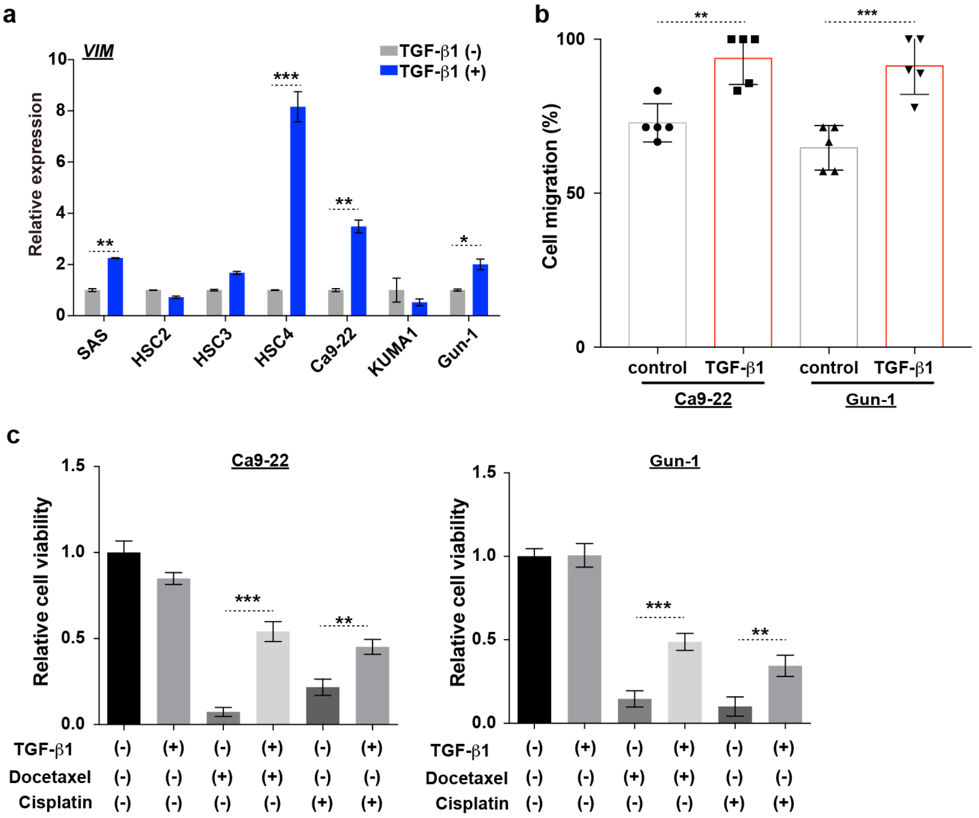

Supplementary figure 1: TGF-β promoted cell motility and increased chemoresistance in HNSCC cells. (a) RT-qPCR for *VIM* mRNA expression in seven different HNSCC cell lines that were unstimulated or stimulated with 2 ng/ml human recombinant TGF-β1 for 48 h. Each value was normalized to *GAPDH* mRNA expression. **(b)** Wound healing assay of Ca9-22 and Gun-1 cells cultured with or without 2 ng/ml TGF-β1 for 48 h. Cell migration distances after 10 h at five randomly chosen points were measured and compared with the distances at 0 h. **(c)** Cell viability assays in TGF-β1-unstimulated or -stimulated Ca9-22 (left) and Gun-1 (right) cells that were exposed to DMSO, docetaxel, or cisplatin. The data are represented as mean ± SEM. All *p*-values were determined by Student's *t* test. *p<0.05, **p<0.01, ***p<0.001.

Supplementary figure 2

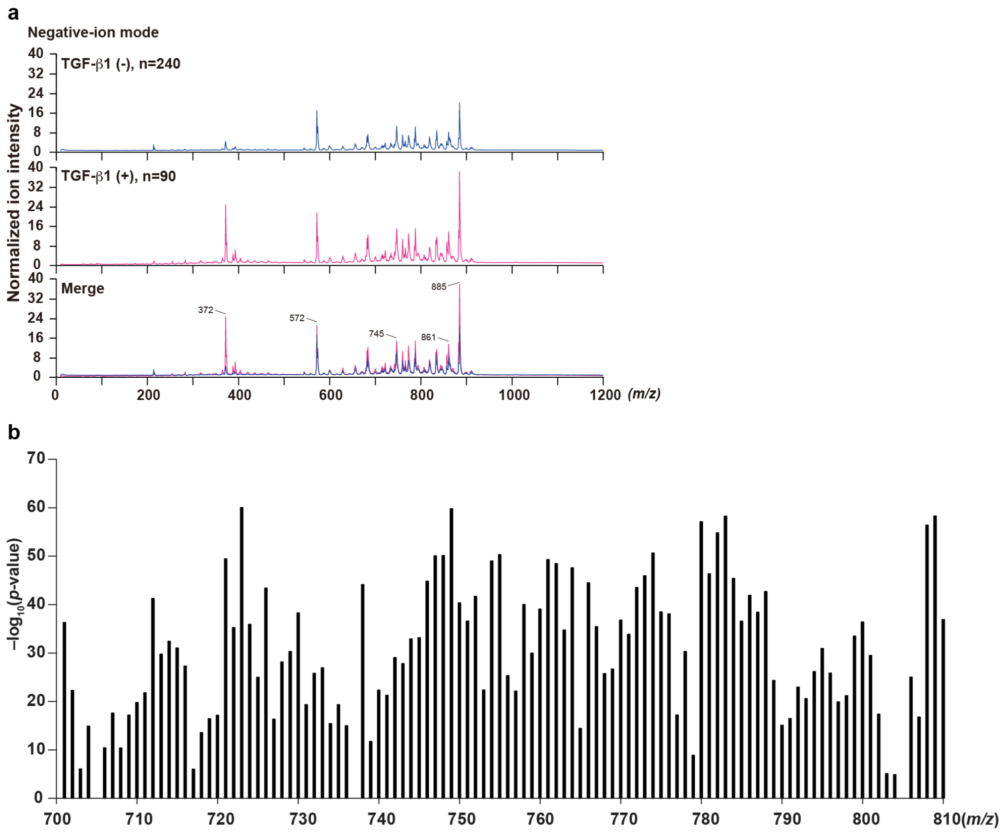

Supplementary figure 2: Construction of a discriminant algorithm to detect activation of TGF-β signaling in HNSCC cells. (a) Mean normalized mass spectra (*m/z* 10–1200) of HNSCC cells that were unstimulated (upper: n=240) or stimulated with 2 ng/ml TGF-β1 for 48 h (middle: n=90) in negative-ion mode. Merged mass spectra are shown in the bottom panel for comparisons between TGF-β1-unstimulated and -stimulated HNSCC cells. (**b**) The $-\log_{10}$(*p*-value) for each *m/z* value in positive-ion mode.

Supplementary figure 3

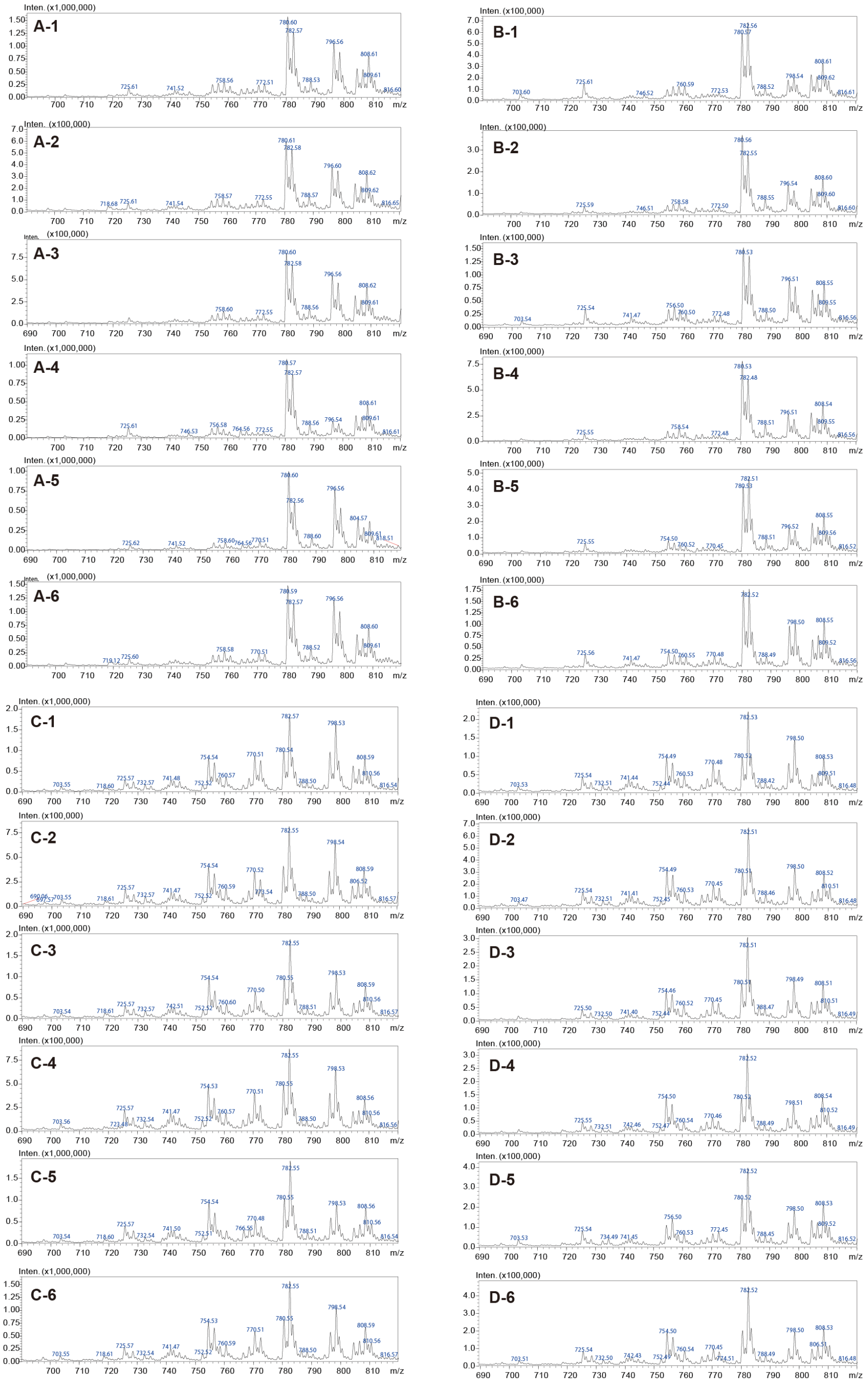

Supplementary figure 3: ***In vivo*** **lipid metabolome-based rapid diagnosis of tumor areas with activated TGF-β signaling in human HNSCC tissues.** Mass spectra data of tumor specimens from a central or marginal area of HNSCC tissues.

Supplementary figure 4

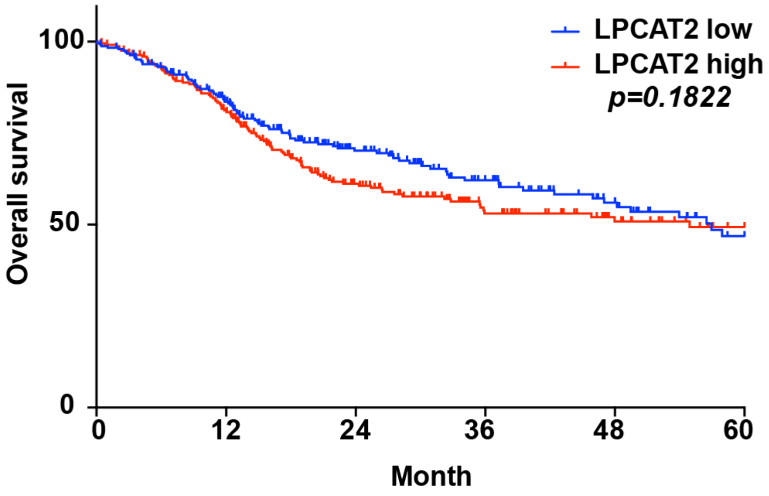

Supplementary figure 4: *LPCAT2* correlates with lymph node metastases and decreased survival in HNSCC patients. Overall survival in HNSCC patients with low or high expression of LPCAT2. The TCGA data were used.
